# Mechanistic Artificial Intelligence for Personalized Drug Therapy: Integrating Pharmacokinetics, Pharmacodynamics, Therapeutic Drug Monitoring, and Multiomic Systems Biology

**DOI:** 10.3390/pharmaceutics18081012

**Published:** 2026-08-16

**Authors:** Ian Jenkins, Krista Casazza, Waldemar Lernhardt, Valentina Savich, Jayson Uffens, Vaishnavi Narayan, Jonathan R. T. Lakey

**Affiliations:** 1 GATC Health Corp., Irvine, CA 92614, USA; ian@gatchealth.com (I.J.); waldemar@gatchealth.com (W.L.); valentina@gatchealth.com (V.S.); jayson@gatchealth.com (J.U.); vaish@gatchealth.com (V.N.); 2 Departments of Surgery and Biomedical Engineering, University of California Irvine, Irvine, CA 92868, USA; krista.casazza@gmail.com

**Keywords:** therapeutic drug monitoring, model-informed precision dosing, pharmacokinetics, pharmacodynamics, precision medicine, systems pharmacology, mechanistic artificial intelligence, multiomics, pharmacogenomics, personalized drug therapy

## Abstract

Interindividual variability in drug response remains a major challenge in clinical pharmacology despite substantial advances in therapeutic drug monitoring (TDM), pharmacogenomics, pharmacokinetics/pharmacodynamics (PK/PD), and model-informed precision dosing (MIPD). Although these approaches have improved individualized therapy, clinically important variability in efficacy and toxicity persists because drug response is determined not only by systemic exposure but also by target engagement, disease biology, compensatory pathways, organ function, immune status, and dynamic patient-specific molecular states. Recent advances in multiomics, systems pharmacology, and artificial intelligence (AI) provide an opportunity to integrate these complementary biological and clinical dimensions within more comprehensive precision pharmacotherapy frameworks. This narrative review examines the evolving integration of PK, PD, TDM, pharmacometrics, multiomic technologies, mechanistic AI, and systems pharmacology across drug development and clinical care. Particular emphasis is placed on the limitations of exposure-based dosing alone, the biological determinants of interindividual variability, the transition from conventional TDM toward adaptive model-informed monitoring, and emerging approaches for integrating molecular and clinical data to support individualized therapeutic decision-making. Operon™ is discussed as an illustrative example of an internally operated mechanistic systems biology platform to demonstrate how biologically informed computational frameworks may integrate pharmacological and multiomic information within drug development workflows. The review further examines applications in polypharmacy, drug–drug interaction assessment, clinical trial enrichment, regulatory science, and adaptive dosing, while emphasizing that analytical validity, clinical validity, clinical utility, prospective validation, transparency, and clearly defined contexts of use remain essential prerequisites for clinical implementation. Collectively, these developments support a transition from concentration-guided dosing toward mechanism-informed precision pharmacotherapy that integrates drug exposure with biological response and clinical outcomes while maintaining rigorous standards for validation and regulatory acceptance.

## 1. Introduction

Personalized drug therapy seeks to optimize treatment selection and dosing for the individual patient by integrating pharmacokinetic (PK) and pharmacodynamic (PD) principles, therapeutic drug monitoring (TDM), pharmacogenomics, and patient-specific characteristics such as age, body composition, organ function, comorbidities, and concomitant medications. These approaches are particularly established for drugs with narrow therapeutic windows, substantial interindividual PK variability, or clinically meaningful exposure–response relationships, including selected antimicrobials, immunosuppressants, antiepileptics, anticoagulants, and oncology agents [1,2,3]. However, the expanding complexity of modern pharmacotherapy exposes the limitations of conventional personalization [4,5]. Consequently, dose, plasma concentration, and inherited genotype alone may not fully explain interindividual differences in efficacy or toxicity, particularly when functional metabolic phenotype and target-pathway activity change over time [6].

The emergence of model-informed drug development (MIDD), model-informed precision dosing (MIPD), physiologically based pharmacokinetic (PBPK) modeling, population PK/PD modeling, and Bayesian forecasting has created a more quantitative foundation for individualized dosing. MIPD extends conventional TDM by combining measured drug concentrations with mathematical models and patient-specific covariates to estimate individual PK parameters and predict optimal dosing regimens [2,3,7]. Regulatory momentum is also increasing: the International Council for Harmonization (ICH) M15 guideline on MIDD provides harmonized principles for planning, evaluating, documenting, and communicating model-informed evidence in drug development [8]. Nevertheless, current PK/PD and TDM frameworks remain incomplete for complex chronic disease [1]. Traditional dosing models often focus on systemic exposure rather than tissue exposure, pathway engagement, disease-state biology, immune-metabolic context, or long-term adaptive responses [9]. Although pharmacogenomics has generated clinically actionable advances in areas such as CYP-mediated metabolism, HLA-associated hypersensitivity, fluoropyrimidine toxicity, and anticoagulant dosing, many drug-response phenotypes are inherently polygenic and dynamically regulated [10]. Genomic variants interact with epigenetic modifications, transcriptional activity, protein abundance, metabolite networks, microbiome-derived signaling, and environmental exposures, creating a biological landscape that cannot be adequately represented by static genotype information alone [11]. Recent evidence emphasizes that integrating genomics, transcriptomics, epigenomics, proteomics, and metabolomics provides a more comprehensive characterization of patient-specific drug response biology and can substantially improve prediction of efficacy and toxicity compared with single-layer approaches [12,13].

**Figure 1:**
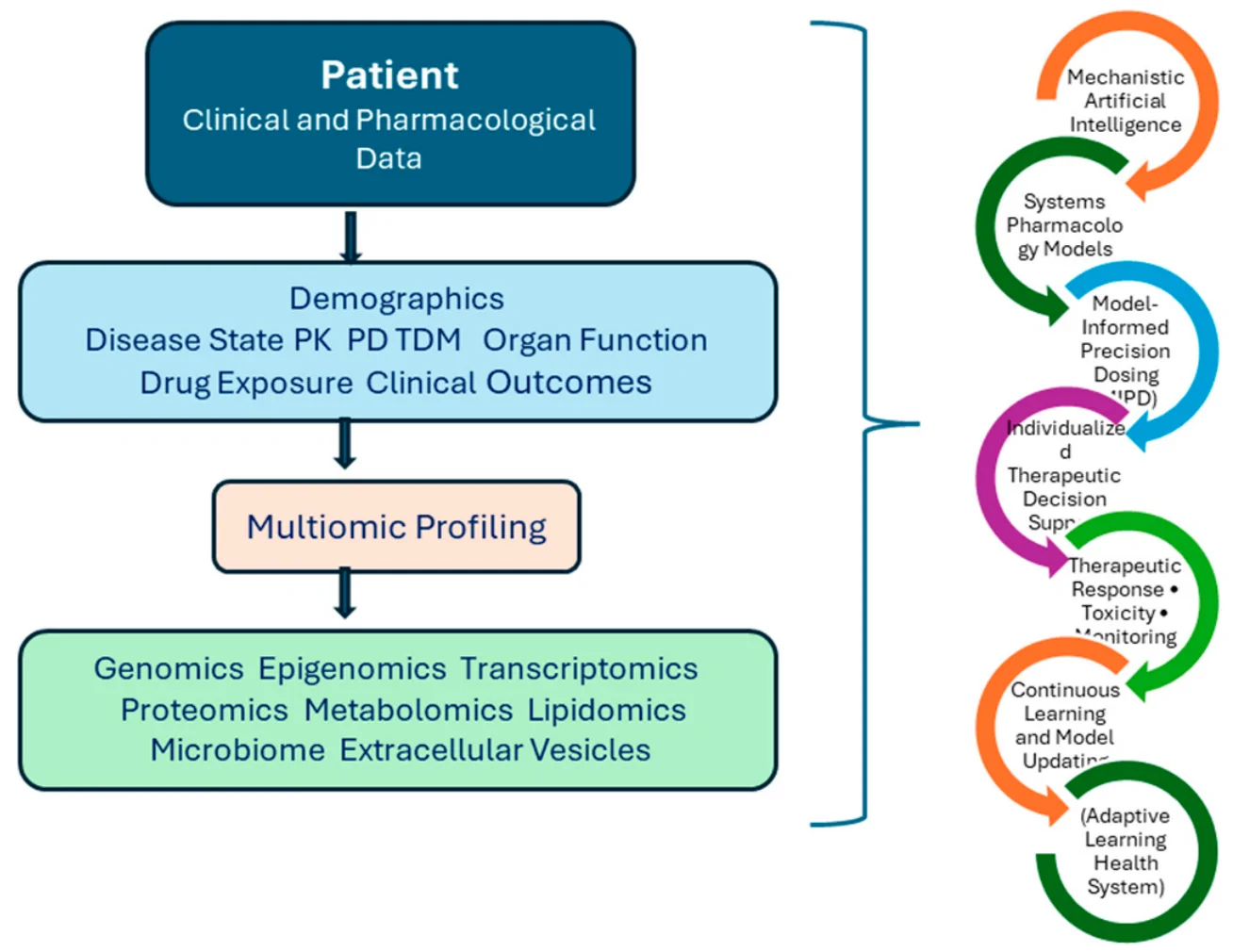
Mechanistic framework for AI-enabled personalized drug therapy.

Recent advances in multiomics, systems pharmacology, and AI are reshaping precision pharmacotherapy by enabling integration of molecular, pharmacological, and clinical information across multiple biological scales. These advances provide an opportunity to extend conventional PK/PD and TDM frameworks beyond exposure-based dosing toward biologically informed therapeutic decision-making. We also consider the relevance of internally operated mechanistic AI platforms, such as Operon™, for therapeutic discovery, PK/PD simulation, off-target risk assessment, and structured expert-reviewed decision support. The central thesis is that the next generation of personalized drug therapy will require a shift from concentration-guided dosing alone toward mechanism-informed, dynamically updated models that link exposure to biological response and clinically meaningful outcomes. The review emphasizes evidence supporting clinical utility, biological plausibility, validation requirements, and regulatory considerations for implementation of AI-augmented precision pharmacotherapy (Figure 1).

Literature Search Strategy

This narrative review synthesized peer-reviewed literature identified through structured searches of the evolving role of mechanistic artificial intelligence (AI), pharmacometrics, TDM, model-informed precision dosing (MIPD), multiomics, and systems pharmacology in personalized drug therapy. Although not performed as a formal systematic review, a structured literature search was undertaken to identify contemporary evidence and landmark publications relevant to precision pharmacotherapy. Literature searches were performed using PubMed/MEDLINE, Embase, Scopus, Web of Science, Google Scholar, and regulatory guidance repositories maintained by the U.S. Food and Drug Administration (FDA), European Medicines Agency (EMA), and ICH. The primary search period encompassed January 2015 through June 2026, although seminal earlier publications were included when considered foundational to therapeutic drug monitoring, pharmacometrics, pharmacogenomics, or systems pharmacology. Search terms included the following combinations: therapeutic drug monitoring, model-informed precision dosing, pharmacometrics, pharmacokinetics, pharmacodynamics, pharmacogenomics, multiomics, systems biology, systems pharmacology, artificial intelligence, machine learning, drug development, digital twins, precision medicine, drug–drug interactions, drug safety, and clinical decision support. Priority was given to recent peer-reviewed original investigations, consensus guidelines, regulatory documents, high-impact reviews, validation studies, and methodological publications addressing clinical implementation. Additional references were identified through citation chaining and review of reference lists from key publications. Because the objective of this review was to provide a forward-looking synthesis of emerging technologies rather than a quantitative meta-analysis, studies were selected based on scientific relevance, methodological rigor, translational significance, and applicability to personalized drug therapy across the pharmaceutical development and clinical care continuum.

## 2. Limitations of Traditional TDM

TDM has been most successful for drugs with narrow therapeutic indices, substantial interindividual pharmacokinetic variability, well-defined exposure–response relationships, and validated therapeutic concentration ranges. These include vancomycin, aminoglycosides, calcineurin inhibitors, lithium, digoxin, selected antiepileptic drugs, and certain antifungal agents. In these settings, TDM improves target attainment, reduces toxicity, and supports dose adjustment in response to altered drug clearance, organ dysfunction, uncertain adherence, and clinically significant drug–drug interactions [1,6,14]. However, conventional TDM is not synonymous with personalized pharmacotherapy. Rather, it is primarily an exposure-management strategy that estimates systemic drug concentrations and only indirectly reflects the biological processes ultimately responsible for therapeutic efficacy or toxicity [2].

First, plasma concentration is an imperfect surrogate for pharmacological effect. A measured concentration may approximate systemic exposure, but the clinically relevant exposure may reside in a tissue compartment, intracellular space, infection site, tumor microenvironment, central nervous system, kidney, liver, or immune-cell population. For antibiotics, the relationship between exposure and microbiological effect depends not only on host PK but also on pathogen susceptibility, inoculum burden, site penetration, immune function, and the relevant PK/PD index, such as AUC/Minimum Inhibitory Concentration (MIC), Cmax/MIC, or time above MIC [15]. For example, the 2020 vancomycin consensus guideline illustrates this evolution. In the guideline, trough-only monitoring was de-emphasized because it was an imperfect surrogate for AUC and was associated with higher nephrotoxicity, while AUC-guided dosing was recommended to better align exposure with efficacy and safety [16]. This example demonstrates a broader principle, i.e., clinically meaningful monitoring requires the correct exposure metric and biological context, not merely a drug level.

Second, traditional TDM frequently relies on sparse sampling, steady-state assumptions, delayed laboratory turnaround, and manual interpretation. These features are poorly aligned with dynamic clinical states such as sepsis, acute kidney injury, extracorporeal support, critical illness, transplantation, oncology, or rapidly changing inflammatory conditions. In such settings, drug clearance and volume of distribution can change over hours to days, making single concentrations difficult to interpret without a model that accounts for time-varying physiology. Emerging TDM technologies, including point-of-care assays, biosensors, and continuous or minimally invasive monitoring approaches, may improve temporal resolution, but their clinical value depends on integration with PK/PD models and decision frameworks rather than measurement alone [2,7].

Third, TDM generally answers whether exposure is within a predefined range, but not whether the drug is producing the intended molecular effect or whether compensatory pathways are reducing efficacy. This limitation becomes increasingly important as therapeutics target complex molecular networks rather than single enzymes or receptors. Drug response is often influenced by dynamic changes in gene expression, epigenetic regulation, protein signaling networks, metabolic adaptation, and host–microbiome interactions. Multiomic investigations have repeatedly demonstrated that clinically relevant variation in treatment response may arise from biological mechanisms that are not detectable through drug concentration measurements alone [12,13]. Consequently, exposure-guided monitoring without mechanistic biological context may underestimate emerging resistance, adaptive pathway activation, or impending toxicity. For many modern therapies, i.e., biologics, kinase inhibitors, immunomodulators, gene-targeted agents, and metabolic drugs, clinical response depends on target expression, pathway activation, immune context, tissue penetration, receptor occupancy, downstream signaling, and disease heterogeneity. In these cases, concentration without PD or systems context may be insufficient. Two patients with similar exposure may have different efficacy due to target biology, and two patients with different exposure may have similar outcomes if pathway sensitivity differs.

Fourth, TDM is often drug-centric rather than regimen-centric. Actual patients frequently receive multiple therapies that interact through absorption, metabolism, transport, protein binding, renal elimination, hepatic clearance, QT liability, immune modulation, hemodynamics, and overlapping toxicity pathways [5]. A TDM result for a single drug may not capture the cumulative risk arising from an entire regimen. Moreover, modern pharmacotherapy increasingly involves interactions across biological networks rather than isolated pharmacological targets. System-level analyses reveal that drug response frequently emerges from perturbations of interconnected signaling, metabolic, inflammatory, and regulatory pathways [5,17]. This complexity is particularly relevant in patients receiving multiple therapies, where cumulative biological effects may not be reflected in the concentration of any single agent. Multiomic layers may also provide direct biological context for PK and PD variability. Transcriptomic and proteomic signatures can reflect real-time enzyme and transporter activity more accurately than genotype alone. Metabolomic profiles may identify mitochondrial dysfunction, altered hepatic metabolic capacity, oxidative stress, or renal injury before conventional laboratory abnormalities emerge. Extracellular vesicle (EV) cargo may provide compartment-specific information regarding tissue injury, target engagement, or treatment resistance [18,19]. Multiomic and network-based approaches therefore offer an opportunity to evaluate therapeutic regimens as integrated biological systems rather than collections of independent drugs. This limitation is especially important in older adults, transplant recipients, oncology patients, patients with cardiovascular–kidney–metabolic disease, and individuals with polypharmacy. Personalized therapy increasingly requires modeling of the therapeutic system, not simply monitoring one analyte [20].

Fifth, many therapeutic drug monitoring target ranges are derived from population-based exposure–response relationships and therefore may not represent the optimal therapeutic window for an individual patient. Therapeutic ranges generally reflect concentrations associated with acceptable efficacy and toxicity in populations rather than individualized biological responses. They do not fully account for pharmacogenomic variation, disease severity, longitudinal changes in organ function, inflammation-mediated phenoconversion, obesity-related alterations in drug distribution, pregnancy, renal replacement therapies, microbiome-mediated metabolism, or competing clinical priorities [9,11,]. Model-informed precision dosing (MIPD) addresses part of this limitation by integrating population pharmacokinetic models, Bayesian estimation, measured drug concentrations, and patient-specific covariates to generate individualized dosing recommendations [2,21]. Nevertheless, implementation remains inconsistent because of challenges related to model qualification, software usability, electronic health record integration, clinician education, reimbursement, institutional governance, and the availability of prospective clinical evidence demonstrating improved patient outcomes [8,21].

Finally, in many therapeutic drug monitoring applications, analytical capability has advanced more rapidly than evidence demonstrating that concentration-guided monitoring consistently improves clinically meaningful outcomes. Measurement alone is insufficient; individualized dosing strategies should demonstrate improvements in efficacy, toxicity reduction, treatment durability, hospitalization, cost-effectiveness, or patient-centered outcomes. Recent implementation frameworks therefore emphasize rigorous analytical validation, clinical validation, model qualification, prospective evaluation, and clearly defined contexts of use before widespread clinical adoption [8,21,22]. Similarly, reporting guidelines for artificial intelligence-based prediction models, including TRIPOD+AI, CONSORT-AI, and SPIRIT-AI, underscore the importance of transparent reporting of missing data, calibration, uncertainty, subgroup performance, intended clinical use, and external validation rather than relying solely on measures of discrimination [23,24,25]. These limitations do not diminish the value of therapeutic drug monitoring; rather, they define the next stage of its evolution. Conventional TDM established the principle that measured drug exposure can guide therapy. The emerging challenge is therefore to integrate drug exposure with patient physiology, disease biology, multiomic profiles, pharmacodynamic biomarkers, multidrug regimens, and longitudinal clinical outcomes within mechanistically informed precision pharmacotherapy frameworks (Table 1).

**Sources of Interindividual PK Variability**

Interindividual pharmacokinetic (PK) variability remains a major challenge in precision pharmacotherapy because patients receiving identical drug doses frequently achieve markedly different systemic and tissue drug exposures, therapeutic responses, and toxicity profiles. Classical determinants of PK variability—including age, sex, body composition, renal function, hepatic function, gastrointestinal absorption, plasma protein binding, and concomitant medications—remain important but explain only a portion of the variability observed in clinical practice. Increasing evidence indicates that drug absorption, distribution, metabolism, and excretion (ADME) represent dynamic physiological phenotypes influenced by interacting genetic, molecular, microbial, disease-related, and environmental factors that evolve throughout the course of disease and treatment [2,9,]. Among these determinants, variation in drug-metabolizing enzymes and membrane transport systems represents one of the principal sources of exposure heterogeneity. Cytochrome P450 enzymes, UDP-glucuronosyltransferases, sulfotransferases, ATP-binding cassette (ABC) transporters, and solute carrier (SLC) transporters collectively govern the disposition of most therapeutic agents and exhibit substantial interindividual variability [10]. Although pharmacogenomic testing identifies inherited variants affecting enzyme or transporter activity, genotype alone frequently fails to predict observed drug exposure because functional metabolic capacity is continuously modified by inflammation, disease severity, nutritional status, concomitant medications, and environmental exposures. Consequently, the clinically relevant phenotype is often the current activity of the metabolic and transport network rather than inherited genotype alone. A particularly important manifestation of this phenomenon is phenoconversion, whereby the observed metabolic phenotype differs from that predicted by genotype [11]. Inflammatory cytokines, infection, malignancy, obesity, chronic kidney disease, hepatic dysfunction, and enzyme-inhibiting medications can suppress CYP activity sufficiently to convert genetically normal metabolizers into functional intermediate or poor metabolizers, whereas enzyme induction may substantially increase drug clearance and reduce therapeutic exposure [26]. Recognition of phenoconversion underscores the limitations of relying exclusively on static pharmacogenomic information for individualized dosing.

The gut microbiome represents an additional and increasingly recognized determinant of PK variability. Intestinal microbial communities directly metabolize numerous drugs, generate active and inactive metabolites, influence enterohepatic recirculation, modify bile acid metabolism, alter intestinal permeability, and regulate host expression of drug-metabolizing enzymes and transport proteins [27,28]. Through these mechanisms, the microbiome functions as a dynamic metabolic organ capable of influencing both systemic drug exposure and downstream pharmacodynamic responses. Consequently, interindividual variation in microbial composition, antibiotic exposure, diet, and disease state may contribute substantially to variability in therapeutic efficacy and toxicity.

Disease-associated physiological remodeling further complicates prediction of drug disposition. Chronic kidney disease alters renal clearance, protein binding, transporter activity, and nonrenal metabolic pathways [29]. Hepatic dysfunction affects first-pass metabolism, hepatic enzyme activity, biliary elimination, and hepatic blood flow. Heart failure modifies gastrointestinal absorption, renal perfusion, hepatic congestion, and tissue distribution, whereas obesity alters volume of distribution, adipose partitioning, inflammatory signaling, and hepatic metabolic capacity [30]. Because these pathophysiological adaptations evolve over time, drug disposition is inherently dynamic and cannot be fully characterized by fixed dosing algorithms or single baseline measurements. Collectively, these observations support a transition from viewing PK variability as a collection of isolated covariates toward understanding it as a systems-level phenotype emerging from the interactions among enzyme activity, transporter function, microbiome metabolism, organ physiology, disease biology, and therapeutic exposures. This systems perspective provides the biological rationale for integrating pharmacogenomics, multiomic profiling, therapeutic drug monitoring, pharmacometric modeling, and mechanistically informed artificial intelligence within next-generation precision pharmacotherapy frameworks.

**Multiomics as a Foundation for Precision Pharmacotherapy**

Integrated molecular profiling extends pharmacogenomics by providing a multidimensional representation of the biological processes governing drug disposition, target engagement, therapeutic response, and toxicity. Rather than serving solely as a molecular characterization tool, the principal value of multiomics lies in its ability to resolve biological mechanisms underlying interindividual variability in drug response. By integrating complementary layers of biological organization, including genomics, epigenomics, transcriptomics, proteomics, metabolomics, lipidomics, microbiomics, and extracellular vesicle biology, multiomic approaches provide systems-level insight into molecular processes that cannot be inferred from pharmacokinetic measurements alone [13,31]. Traditional therapeutic drug monitoring and pharmacokinetic modeling estimate systemic drug exposure; however, drug concentration alone is an incomplete surrogate for biological effect. Therapeutic response is determined by target abundance, receptor occupancy, intracellular signaling, tissue penetration, immune status, organ reserve, compensatory pathway activation, and susceptibility to toxicity [1,2]. Multiomic profiling provides complementary biological information capable of identifying whether a therapeutic target has been engaged, whether downstream pathways are responding as expected, and whether adaptive or off-target mechanisms are emerging during treatment. Consequently, multiomics has the potential to complement rather than replace conventional PK/PD approaches by providing biological context for exposure–response relationships. Multiomic profiling also has important implications for patient stratification. Individuals with similar clinical diagnoses and comparable systemic drug exposure may differ substantially in target expression, inflammatory state, metabolic capacity, immune function, and susceptibility to adverse effects. Integrated molecular profiling can identify biologically distinct disease endotypes that help explain this heterogeneity and may ultimately support more individualized therapeutic strategies. The greatest clinical experience to date has been obtained in oncology, where integrated genomic, transcriptomic, proteomic, and metabolomic analyses have improved molecular classification, biomarker discovery, therapeutic target identification, and prediction of drug sensitivity or resistance [12,17]. Although these applications have largely been developed in cancer, they illustrate broader biological principles that are increasingly relevant across clinical pharmacology: therapeutic response frequently emerges from interactions among multiple molecular pathways rather than single genetic variants or isolated pharmacokinetic parameters.

Systems-level molecular characterization has expanded opportunities for therapeutic target identification, biomarker discovery, drug repurposing, toxicity prediction, and clinical trial enrichment. Recent reviews have highlighted how integration of multiomic datasets with artificial intelligence and systems pharmacology can improve identification of disease mechanisms, prioritize therapeutic targets, and facilitate biologically informed drug development [17,32]. These advances are consistent with an emerging transition from reductionist “one drug-one target” paradigms toward network-based models that incorporate multiple interacting biological pathways and patient-specific molecular characteristics. Importantly, however, prospective clinical validation remains limited, and further studies are required to establish the clinical utility of multiomic-guided therapeutic decision-making beyond established pharmacogenomic applications. Changes in transcriptomic or proteomic signatures may reflect pathway inhibition before measurable clinical improvement; metabolomic alterations may identify emerging mitochondrial dysfunction or altered substrate utilization; lipidomic remodeling may provide insight into inflammatory or membrane-associated processes; and microbiome-derived metabolites may influence both drug metabolism and immune regulation [18,19,27,31]. These observations support the concept of biology-guided dosing, in which therapeutic optimization considers both systemic drug exposure and the biological response generated by treatment. Nevertheless, translation of multiomic biomarkers into routine clinical practice requires rigorous analytical validation, standardized sample acquisition and processing, reproducible computational pipelines, external validation, and demonstration of clinical utility. High-dimensional datasets remain susceptible to batch effects, missing modalities, tissue specificity, platform heterogeneity, confounding, and limited sample sizes, emphasizing the importance of robust study design and independent validation before widespread implementation.

A practical challenge in multimodal pharmacotherapy is that pharmacokinetic measurements, pharmacodynamic biomarkers, and multiomic datasets are often acquired at different temporal resolutions and biological scales. Drug concentrations may change over hours, whereas transcriptomic, proteomic, or metabolomic responses evolve over days or weeks and may vary among tissues. Consequently, successful integration requires explicit temporal alignment, handling of missing modalities, uncertainty propagation, and validation that biological measurements correspond to clinically relevant pharmacological states. Prospective longitudinal datasets incorporating synchronized PK, PD, and molecular measurements will be essential for development and validation of multimodal precision dosing frameworks (Table 2).

**MIPD and Pharmacometrics**

MIPD represents the quantitative bridge between TDM and individualized pharmacotherapy. Whereas traditional TDM often follows a measure-and-adjust logic, MIPD integrates drug concentrations, population PK or PK/PD models, patient covariates, Bayesian estimation, and exposure targets to simulate individualized dosing regimens. Contemporary definitions describe MIPD as an advanced quantitative approach using mathematical and statistical models of drugs and disease combined with individual patient characteristics to optimize dosing and improve the benefit–harm balance [2,3,7].

Pharmacometrics provides the methodological foundation for MIPD. Population PK models characterize typical drug disposition and between-subject variability; covariate models explain part of that variability using factors such as renal function, body size, age, genotype, albumin, inflammation, or interacting drugs [33]. Bayesian forecasting updates individual parameter estimates using observed concentrations; and PK/PD models link exposure to response or toxicity [2,34]. This framework has been most widely applied in antimicrobials, immunosuppressants, oncology, pediatrics, critical care, and other settings where exposure variability is clinically consequential. MIPD is particularly valuable when the therapeutic window is narrow, toxicity is exposure-related, the dose–response relationship is nonlinear, or patient physiology changes rapidly. The evolution from trough-based monitoring toward exposure-based metrics such as AUC-guided dosing illustrates the broader transition from empirical therapeutic monitoring toward quantitatively informed exposure optimization [16].

**Figure 2:**
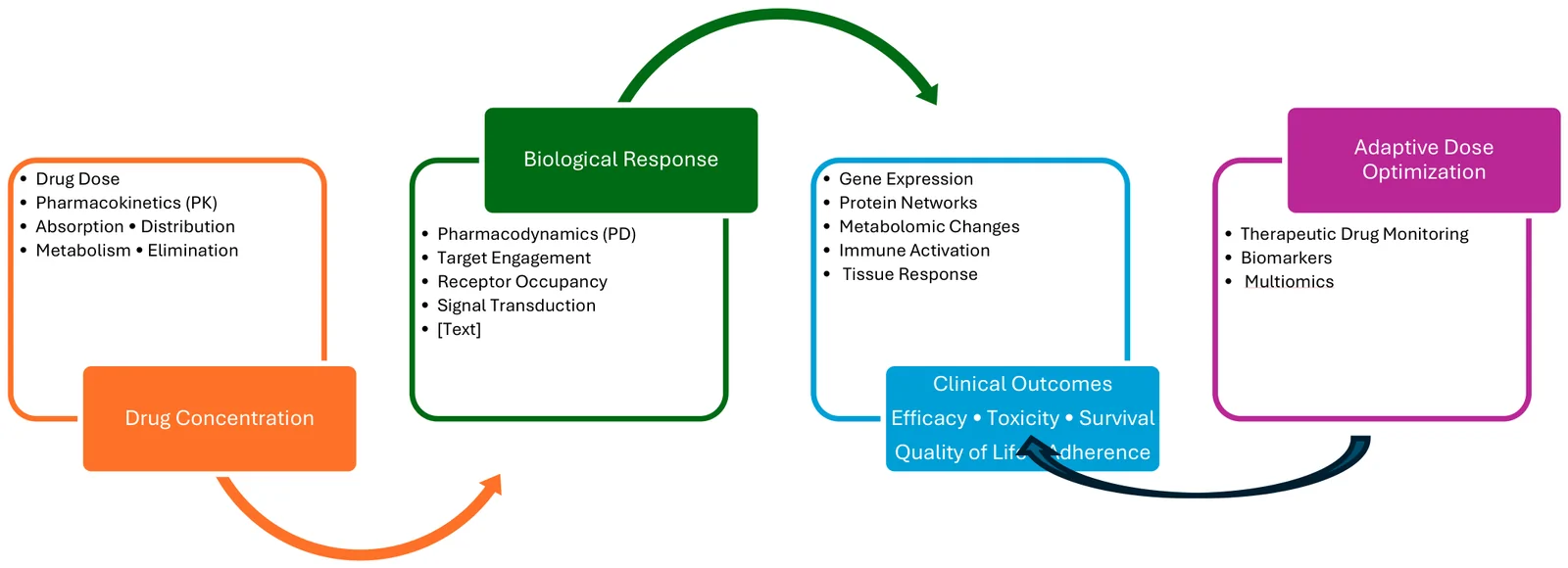
Integration of PK, PD, biological response, and clinical Outcomes. Drug exposure alone is insufficient to explain clinical outcomes. Pharmacodynamic response is modified by molecular, physiological, and disease-specific factors that create feedback between biological response and subsequent therapeutic decisions.

Despite its considerable promise, MIPD has not yet become routine clinical practice across most therapeutic areas. Persistent implementation barriers include limited prospective clinical validation, uncertainty regarding model qualification and selection, lack of standardized software evaluation, workflow integration challenges, interoperability with electronic health records, reimbursement limitations, clinician education, and institutional governance. Recent implementation studies have consistently identified these organizational and technical barriers while emphasizing that availability of pharmacometric models alone is insufficient to ensure successful bedside adoption [3,21]. Similarly, recent evaluations of MIPD software platforms conclude that although these tools facilitate individualized dose optimization, robust prospective evidence demonstrating improvements in clinically meaningful patient outcomes remains limited for many therapeutic indications [35]. Although MIPD provides individualized dose optimization using pharmacometric models and patient-specific pharmacokinetic data, it generally relies on predefined structural models of drug disposition and exposure–response relationships. Mechanistically informed artificial intelligence extends this framework by incorporating biological networks, multiomic data, and systems-level representations of disease biology that are not explicitly represented in conventional pharmacometric models (Figure 2).

**Mechanistic AI and Systems Pharmacology**

Mechanistic AI refers to computational approaches that explicitly incorporate biological knowledge, causal pathway relationships, and systems-level constraints to model how drug exposure perturbs molecular networks and ultimately produces therapeutic efficacy or toxicity. Unlike purely predictive AI, which identifies statistical associations to estimate clinical outcomes, mechanistic AI integrates established biological knowledge with data-driven inference to generate biologically interpretable hypotheses that can be independently evaluated experimentally. Mechanistic AI therefore emphasizes causal biological representation in addition to predictive performance [36,37,38].

As precision pharmacotherapy evolves beyond exposure-guided dosing, therapeutic response is increasingly recognized as an emergent property of interactions among drug exposure, target biology, disease mechanisms, compensatory signaling pathways, organ-system physiology, and patient-specific biological context. Although pharmacometric modeling and model-informed precision dosing remain highly effective for characterizing concentration-time relationships and optimizing dose selection, many contemporary therapeutic challenges involve nonlinear biological processes that are not explicitly represented in conventional PK/PD models [2,36]. These challenges have stimulated increasing interest in mechanistically informed AI and systems pharmacology approaches capable of integrating molecular, physiological, and clinical information across multiple biological scales. Predictive AI, mechanistic AI, systems pharmacology, and model-informed precision dosing represent complementary rather than competing computational paradigms (Table 3). Predictive AI focuses primarily on outcome prediction, risk stratification, or classification by identifying statistical relationships within large clinical datasets [39]. Such models may accurately predict therapeutic response or adverse events without explicitly identifying the biological mechanisms responsible for those predictions. Mechanistic AI extends this framework by incorporating biological pathways, molecular interactions, and physiological constraints that permit interpretation of *why* a particular therapeutic response occurs [36,37]. In pharmacology, this distinction is clinically important because therapeutic decisions often depend upon understanding the underlying mechanism of treatment failure or toxicity. Reduced efficacy resulting from inadequate drug exposure requires different intervention than treatment failure caused by pathway redundancy, altered receptor biology, inflammatory phenoconversion, or adaptive network remodeling. Systems pharmacology provides the conceptual framework within which mechanistic AI operates, viewing drug response as an emergent property of interconnected biological systems rather than isolated molecular targets [40]. A fundamental limitation of exposure-centric pharmacological models is the assumption that similar systemic drug exposure produces similar biological effects. Therapeutic response varies because target abundance, receptor occupancy, intracellular signaling, immune activation, metabolic reserve, tissue penetration, and compensatory biological pathways differ substantially among patients. Systems pharmacology therefore complements conventional PK/PD models by integrating these biological determinants of response within network-based representations of disease and pharmacology rather than replacing established pharmacometric methods [41].

Recent advances in AI have substantially expanded the ability to model this biological complexity. Graph neural networks, transformer architectures, multimodal learning frameworks, and other network-based approaches can integrate heterogeneous datasets spanning genomics, transcriptomics, proteomics, metabolomics, medical imaging, electronic health records, and real-world clinical outcomes [32]. Because biological systems are inherently organized as interacting molecular networks, graph-based approaches are particularly well-suited for representing relationships among genes, proteins, metabolites, signaling pathways, therapeutic agents, and adverse events. These methods have increasingly been applied to identify therapeutic targets, predict off-target effects, characterize disease mechanisms, prioritize drug repurposing opportunities, and model biological responses to pharmacological perturbation [12,32].

Mechanistically informed AI also has applications throughout the drug development continuum. Integration of multiomic and clinical datasets may support target identification, biomarker discovery, toxicity prediction, ADMET modeling, patient stratification, clinical trial enrichment, and drug repurposing [42]. Emerging computational frameworks further permit simulation of alternative therapeutic strategies within biologically constrained network models before experimental evaluation. Importantly, mechanistic AI should be viewed as an extension of established quantitative pharmacology rather than a replacement for it. Pharmacokinetic models quantify drug exposure, pharmacodynamic models characterize exposure–response relationships, model-informed precision dosing individualizes dose selection, systems pharmacology represents biological network behavior, and mechanistic AI integrates these complementary components within biologically interpretable computational frameworks capable of supporting individualized therapeutic decision-making. Ultimately, the clinical value of these approaches will depend not only on predictive accuracy but also on prospective demonstration that they improve therapeutic efficacy, safety, clinical decision-making, or cost-effectiveness relative to existing standards of care [8,23,25] (Table 3).

**Operon™ as a Systems Platform for PK/PD Integration**

A mechanistic AI platform for personalized drug therapy must integrate PK measurements, PD biomarkers, multiomic biology, clinical phenotypes, real-world outcomes, and prior biological knowledge into a coherent decision environment. Operon is presented as one example of an internally operated mechanistic systems-biology platform illustrating how AI may be integrated within contemporary PK/PD workflows. The platform is discussed to demonstrate conceptual implementation rather than clinical performance and should not be interpreted as evidence of clinical superiority over existing approaches. Operon™ is an internally operated, AI-driven systems biology platform developed to support therapeutic discovery and development by integrating PK, PD, multiomic, and clinical data within biologically informed analytical workflows. The distinguishing feature of a mechanistic platform is not simply predictive performance but biological traceability. Importantly, the scientific value of any mechanistic platform ultimately depends on prospective validation of generated hypotheses and demonstration that platform-derived insights improve decision quality beyond existing pharmacological approaches. Rather than treating molecular variables as independent predictors, mechanistic architectures attempt to preserve known biological relationships among genes, proteins, pathways, tissues, and physiological processes. This enables hypotheses regarding why a particular efficacy, safety, or exposure outcome is predicted, thereby supporting scientific review, experimental validation, and translational decision-making.

Notwithstanding, platform-derived predictions require validation for each context of use. Performance in one domain, endpoint, or dataset does not guarantee performance in another. For PK/PD integration, validation should include analytical reproducibility, biological plausibility, retrospective external validation, prospective target-attainment studies, exposure–response validation, toxicity prediction performance, calibration across subgroups, and clinical utility endpoints. The Biomarker Research review emphasizes that AI models may be biased if trained on unrepresentative datasets and that reliance on historical or synthetic data can lead to overfitting or poor generalizability. The potential value of Operon™ lies in making this integration operational: transforming fragmented data into structured hypotheses, risk assessments, and development strategies that can be reviewed by pharmacologists, clinicians, and translational scientists. The ultimate test will be prospective evidence that such systems improve dosing precision, therapeutic response, toxicity avoidance, and decision efficiency relative to conventional approaches. Because platform performance is context-dependent, any Operon™-derived PK/PD or dosing hypothesis should be validated against the specific endpoint, population, drug class, and intended decision before clinical implementation. Any AI-augmented platform intended to support PK/PD interpretation, therapeutic monitoring, or treatment decision support should be evaluated within a clearly defined context of use and according to contemporary regulatory expectations regarding model credibility, validation, transparency, and lifecycle governance.

**AI-augmented TDM**

The principal distinction between conventional MIPD and AI-augmented TDM is the ability to learn complex, nonlinear relationships from large-scale multimodal datasets that extend beyond traditional PK covariates. Classical MIPD frameworks generally rely on predefined structural models incorporating factors such as body size, age, renal function, hepatic function, genotype, and measured drug concentrations [2,3,7]. These approaches have demonstrated substantial value, particularly for narrow-therapeutic-index drugs, but their predictive performance may be limited when clinically relevant determinants of drug exposure and response are incompletely measured, highly interactive, or evolve over time.

AI methods can augment, rather than replace, pharmacometric modeling by incorporating heterogeneous data streams that are difficult to represent within conventional population PK frameworks. These include longitudinal electronic health record (EHR) data, laboratory trajectories, medication histories, clinical notes, physiologic monitoring, wearable-device outputs, pharmacogenomic information, and emerging multiomic biomarkers. Machine-learning approaches can identify nonlinear interactions among these variables and recognize latent patterns that may not be captured by predefined covariate structures, associated with changes in drug clearance, target engagement, toxicity susceptibility, adherence behavior, and disease progression [32,39,43].

One promising application is dynamic therapeutic monitoring. Conventional TDM schedules are often predefined according to population-based assumptions regarding sampling frequency and steady-state timing [34]. AI-augmented systems may support adaptive measurement strategies by estimating when additional concentration measurements are most likely to reduce uncertainty or influence clinical decisions. Such approaches could reduce unnecessary laboratory testing while prioritizing monitoring during periods of physiological instability, medication changes, organ dysfunction, or elevated toxicity risk [44]. Integration of longitudinal clinical data represents another important opportunity. Drug exposure and therapeutic response evolve continuously as disease severity, organ function, inflammatory status, concomitant medications, and clinical interventions change over time. Rather than interpreting isolated concentration measurements, AI-enabled decision-support systems may integrate serial laboratory results, hospitalization history, medication modifications, adverse-event reports, and disease-specific clinical outcomes to provide continuously updated estimates of therapeutic response and risk. Such longitudinal learning frameworks have the potential to transform therapeutic drug monitoring from a static measurement process into an adaptive clinical decision-support system.

Reliable clinical implementation also requires explicit characterization of model uncertainty. AI-based dosing recommendations should communicate prediction intervals, confidence estimates, important model assumptions, missing-data effects, and the degree to which recommendations are supported by available evidence [45]. Transparent uncertainty reporting is particularly important when predictions are generated for patient populations underrepresented in training data or when clinically important variables are unavailable. Similarly, unlike conventional pharmacometric models that typically remain stable over time, machine-learning models require ongoing lifecycle management to detect performance drift resulting from changes in patient populations, prescribing practices, laboratory assays, or standards of care. Continuous monitoring, recalibration, external validation, and prospective clinical evaluation are therefore essential components of trustworthy AI deployment [23,24,25]. Ultimately, the value of AI-augmented TDM should not be judged solely by improvements in predictive accuracy but by its ability to improve clinically meaningful outcomes. Relevant measures include achievement of therapeutic exposure targets, reduction in drug-related toxicity, improved treatment persistence, more efficient healthcare resource utilization, enhanced clinician workflow, and better patient-centered outcomes; as with mechanistic AI more broadly, prospective demonstration of clinical utility remains the critical benchmark for successful implementation.

**Polypharmacy, Drug–Drug Interactions (DDI), and Complex Disease**

Polypharmacy is one of the most clinically important and under-modeled challenges in personalized drug therapy. Patients with chronic diseases commonly receive multiple medications with overlapping metabolic pathways, transporter dependencies, PD effects, and toxicity liabilities. Conventional drug–drug interaction (DDI) assessment often emphasizes pairwise interactions, particularly CYP inhibition/induction, transporter effects, QT prolongation, renal clearance competition, or additive bleeding and sedation risk [8]. While this framework is clinically useful, it is increasingly insufficient for complex disease contexts where the relevant pharmacological unit is the entire regimen rather than any individual drug pair. Interindividual DDI risk is shaped by both PK and PD interactions. PK DDIs alter exposure through effects on absorption, gastric pH, intestinal and hepatic enzymes, efflux and uptake transporters, protein binding, renal secretion, or biliary excretion [46]. PD DDIs alter response without necessarily changing concentrations, through convergent effects on hemodynamics, coagulation, immune activation, glucose homeostasis, cardiac electrophysiology, cognition, mitochondrial function, or renal perfusion. Recent PK/PD modeling literature emphasizes that integrating PK and PD models provides a more reliable framework for evaluating and optimizing drug regimens in DDI research than exposure-only assessment [47,48].

Complex chronic disease amplifies DDI risk because disease biology modifies the same pathways that drugs use. CKD alters renal clearance and protein binding but also changes nonrenal metabolism through uremic toxins and inflammatory signaling. Heart failure modifies hepatic blood flow, gut edema, renal perfusion, and neurohormonal state. Liver disease alters first-pass metabolism, biliary excretion, albumin, and coagulation. Obesity modifies distribution volume, inflammatory tone, hepatic steatosis, and enzyme expression. Cancer, infection, autoimmune disease, and critical illness can induce phenoconversion through cytokine-mediated suppression of CYP enzymes and transporters [49,50]. In this setting, a DDI that appears modest in healthy-volunteer studies may become clinically important in a patient with organ dysfunction, inflammation, or multimorbidity. AI is well-suited to polypharmacy because adverse outcomes often emerge from high-order interactions that are difficult to capture using conventional pairwise rules. Knowledge graphs can represent relationships among drugs, targets, pathways, enzymes, transporters, adverse events, diseases, and patient characteristics. Graph neural networks can infer interaction risk by learning network topology and molecular features. Natural language processing can extract adverse-event signals from EHR notes, pharmacovigilance databases, and literature. These methods may also facilitate extraction of adverse-event and interaction signals from large pharmacovigilance and clinical datasets [51]. Recent reviews of AI-augmented DDI research highlight growing use of knowledge graphs, explainable AI, and multimodal data integration to improve detection and prediction of high-risk combinations [40,52].

Polypharmacology is the intentional design or selection of agents that modulate multiple targets or pathways. In complex diseases such as cancer, infection, diabetes, cardiovascular–kidney–metabolic disease, autoimmune disorders, and neurodegeneration, single-target therapy may be insufficient because disease networks contain redundancy, compensation, and adaptive resistance. Rational polypharmacology seeks to modulate network states deliberately while minimizing cumulative toxicity. Recent work on AI-driven polypharmacology argues that combination regimens can improve therapeutic robustness but also increase DDI risk, cumulative toxicity, adherence burden, and dosing complexity, underscoring the need for AI-assisted design and safety evaluation [53]. Multiomics can improve regimen-level assessment by revealing how combined therapies perturb biological networks. Clinically useful AI systems for polypharmacy should therefore do more than generate alerts [54,55]. Current DDI alerting systems often suffer from alert fatigue because they lack patient-specific context and rank too many theoretical interactions as clinically equivalent. A next-generation system should estimate interaction probability, clinical severity, modifiability, patient-specific susceptibility, uncertainty, and recommended mitigation strategy. It should distinguish mechanistically plausible interactions from low-relevance warnings and incorporate patient factors, such as renal function, genotype, age, frailty, biomarkers, comorbidities, and current drug concentrations. Pharmacists, clinical pharmacologists, and prescribers should remain central because AI-derived recommendations require reconciliation with therapeutic goals, alternatives, patient preferences, and feasibility.

**Applications in Drug Development and Clinical Trials**

AI-augmented PK/PD integration has the potential to transform drug development by addressing many of the fundamental causes of attrition across the pharmaceutical lifecycle, including inadequate target selection, poor efficacy, unexpected toxicity, suboptimal dose selection, and failure to identify responsive patient populations. By integrating multiomic datasets with biological network models, AI can facilitate a transition from reductionist drug discovery toward systems-level therapeutic development. Genomic, transcriptomic, proteomic, metabolomic, microbiomic, single-cell, and spatial datasets can be analyzed jointly to characterize disease-associated networks, prioritize therapeutic targets, identify biomarkers, and generate mechanistically coherent opportunities for drug repurposing or novel therapeutic development.

During candidate discovery and lead optimization, AI-enabled approaches can improve assessment of absorption, distribution, metabolism, excretion, and toxicity (ADMET), binding affinity, off-target effects, synthesizability, and physicochemical properties, thereby accelerating candidate selection while reducing development risk [56]. In clinical development, integration of PK, PD biomarkers, multiomic pathway responses, and safety signals can support selection of biologically optimal doses rather than relying solely on maximum tolerated dose paradigms, an important consideration for targeted therapies, biologics, immunomodulators, and chronic disease interventions where therapeutic benefit may plateau before toxicity thresholds are reached [57]. Such approaches are particularly valuable for therapies characterized by delayed toxicities, immune-mediated mechanisms, tissue-specific activity, or nonlinear exposure–response relationships. AI-driven integration of transcriptomic, proteomic, metabolomic, and clinical data may also improve early detection of hepatotoxicity, cardiotoxicity, nephrotoxicity, immunotoxicity, and other off-target liabilities before large-scale patient exposure, strengthening benefit–risk assessment and reducing late-stage failure. AUC-guided vancomycin dosing, frequently implemented using Bayesian estimation, is associated with lower nephrotoxicity than trough-targeted monitoring while maintaining therapeutic exposure [58,59]. These tools may accelerate candidate prioritization and reduce the number of compounds requiring experimental evaluation. However, computational performance on retrospective or benchmark datasets does not, by itself, establish improved development productivity. Candidate-ranking systems must therefore be evaluated prospectively against experimentally confirmed pharmacological and safety outcomes.

AI-assisted integration of transcriptomic, proteomic, metabolomic, imaging, and clinical data may also support earlier identification of hepatotoxicity, cardiotoxicity, nephrotoxicity, immunotoxicity, and other off-target liabilities [58,59,60]. These applications remain predominantly developmental, however, and require external validation, clinically relevant comparator models, and confirmation that earlier molecular signals improve rather than merely add complexity to benefit–risk assessment. Quantitative pharmacology already provides evidence that model-informed dosing can improve clinically meaningful outcomes. AUC-guided vancomycin dosing, frequently implemented using Bayesian estimation, is associated with lower nephrotoxicity than trough-targeted monitoring while maintaining therapeutic exposure [16,61]. This example demonstrates the clinical value of integrating concentration data with pharmacometric models, although it should not be presented as direct evidence for AI-enabled drug development.

Beyond dose optimization and safety assessment, integrated computational approaches may improve patient stratification and trial enrichment by identifying molecular endotypes, pathway activation states, resistance mechanisms, or PD susceptibility profiles associated with differential treatment response [62]. Biologically informed enrichment may increase observed treatment effects and improve characterization of benefit–risk relationships within defined subgroups. Claims that these approaches necessarily reduce trial sample size or increase the probability of success should remain conditional, because these benefits depend on biomarker prevalence, treatment-effect heterogeneity, assay performance, and the validity of the enrichment strategy.

Synthetic control arms, external comparators, and patient-specific simulation frameworks are also being explored to inform trial feasibility, endpoint selection, dose evaluation, and adaptive study design [63,64].

The term digital twin should be used cautiously because current applications vary widely in biological fidelity, validation, and intended use. In many cases, “patient-specific simulation” or “in silico disease model” is more accurate than implying a continuously synchronized virtual representation of an individual patient. Although AI-enabled approaches may improve the efficiency and biological precision of drug development, implementation depends on high-quality and representative data, control of bias and confounding, transparent model specification, regulatory acceptance, and prospective evidence that the approach improves development or clinical decisions compared with established methods.

**Regulatory Considerations and Validation Requirements**

The increasing integration of AI, MIPD, TDM, and multiomic data into drug development and clinical decision support introduces distinct regulatory and validation requirements. Regulatory acceptance depends not on algorithmic complexity but on whether a model is fit for its intended purpose, produces credible and reproducible outputs, and supports decision-making within a clearly defined context of use. The required level of evidence should be proportional to the influence of the model on regulatory decisions and patient safety [8,65,66,67]. A model used for exploratory target prioritization or hypothesis generation does not require the same evidentiary standard as one used to select a clinical dose, predict severe toxicity, enrich a pivotal trial, or generate patient-specific treatment recommendations. Validation should therefore be risk-based and should address the consequences of an erroneous model output, the degree of human oversight, the availability of confirmatory evidence, and the reversibility of the resulting decision.

The FDA’s 2025 draft guidance[66], Considerations for the Use of Artificial Intelligence to Support Regulatory Decision-Making for Drug and Biological Products, proposes a risk-based credibility assessment framework for AI-generated information submitted in support of drug safety, effectiveness, or quality. The guidance emphasizes the definition of context of use, model risk, data quality, development documentation, performance assessment, and plans for model maintenance. Because it remains draft guidance, the manuscript should describe it as an evolving FDA framework rather than a final binding standard. ICH M15, *General Principles for Model-Informed Drug Development*, reached Step 4 and was adopted in January 2026. It establishes internationally harmonized principles for planning, developing, evaluating, documenting, and applying model-informed evidence across the drug-development lifecycle [8]. M15 addresses MIDD broadly and should not be portrayed as an AI-specific guideline. Rather, it provides the quantitative and regulatory foundation within which AI-enabled pharmacometric models may be evaluated when they are used to support development decisions.

For AI-augmented precision pharmacotherapy, conventional discrimination and prediction metrics, e.g., AUC operating characteristic curve, sensitivity, specificity, calibration, mean prediction error, and root mean square error, are necessary but insufficient. Evaluation should encompass input-data integrity, technical reproducibility, transportability, calibration, subgroup performance, robustness to missing or shifted data, and clinical utility. Analytical validity is most directly applicable when model inputs depend on laboratory, imaging, genomic, or multiomic assays and concerns whether those inputs are measured accurately and reproducibly. Clinical validity concerns whether the model output is reliably associated with the intended PK, PD, efficacy, or safety outcome. Clinical utility requires evidence that use of the system improves therapeutic decisions, patient outcomes, safety, workflow, or resource use relative to an appropriate comparator.

Mechanistic AI requires an additional layer of validation because its outputs may include claims about pathways, targets, causal drivers, or treatment-response mechanisms. These outputs should be evaluated for biological plausibility, consistency with established pharmacology, reproducibility across datasets, sensitivity to alternative model specifications, and concordance with experimental or clinical evidence. A biologically plausible explanation generated by a model is not equivalent to a validated causal mechanism. Unless independently corroborated, such outputs should be described as mechanistic hypotheses rather than causal findings.

Transparency and reporting standards remain essential but should be distinguished from regulatory validation requirements. TRIPOD+AI provides reporting guidance for studies developing or evaluating clinical prediction models using regression or machine-learning methods [25]. CONSORT-AI and SPIRIT-AI provide extensions for reporting clinical trials and trial protocols involving AI interventions [23,24]. These frameworks improve completeness and transparency of reporting, but adherence alone does not establish model credibility, regulatory acceptability, or clinical utility. Lifecycle governance is particularly important for models that continue to learn, are periodically retrained, or depend on clinical data distributions that may change over time. It is also essential to address performance monitoring, drift detection, recalibration, version control, audit trails, cybersecurity, access control, human oversight, and predefined change-management procedures. These safeguards are especially important for AI-augmented TDM and dosing systems because erroneous recommendations may directly alter drug exposure. Multiomic datasets, pharmacogenomic resources, and clinical training cohorts often underrepresent populations defined by ancestry, geography, age, sex, socioeconomic status, disease severity, and comorbidity burden. A model may demonstrate strong aggregate performance while remaining poorly calibrated in clinically important subgroups. Validation should therefore include assessment of discrimination, calibration, error rates, missingness, and clinical consequences across populations relevant to the intended use.

Reproducibility across computational environments further requires documentation of data provenance, preprocessing, feature engineering, model architecture, hyperparameters, software dependencies, versioning, and deployment conditions. Where proprietary constraints limit disclosure, sponsors should still provide sufficient documentation to permit independent evaluation of model behavior and performance. Ultimately, the regulatory future of AI-augmented precision pharmacotherapy will depend on reproducible benefit within clearly defined contexts of use. The evidentiary standard should increase in proportion to the clinical or regulatory consequences of a model-informed recommendation. Systems most likely to achieve adoption will be those that combine pharmacological plausibility, transparent reporting, representative data, prospective validation, lifecycle oversight, and meaningful human clinical review.

**Current Challenges and Research Priorities**

Despite substantial advances in pharmacometrics, multiomics, and AI, several barriers continue to limit the clinical implementation of personalized drug therapy. Most AI-augmented pharmacotherapy models demonstrate promising retrospective performance but lack prospective validation showing improvements in efficacy, safety, target attainment, or patient-centered outcomes. Multiomic technologies remain costly and are not yet routinely integrated into clinical workflows, while assay harmonization, pre-analytical variability, batch effects, and platform-to-platform reproducibility continue to challenge standardization and scalability. Dataset diversity remains another critical concern, as many pharmacogenomic, multiomic, and AI training cohorts underrepresent important ancestral, geographic, socioeconomic, and disease-specific populations, potentially limiting model generalizability and equity. Regulatory pathways for AI-augmented decision support, adaptive dosing systems, and mechanistic modeling platforms continue to evolve, creating uncertainty regarding validation requirements, lifecycle management, and clinical accountability. Future research should prioritize prospective implementation studies, standardized multiomic methodologies, diverse population validation, health-economic assessments, and demonstration that AI-augmented precision pharmacotherapy improves clinically meaningful outcomes beyond existing standards of care (Table 4).

**Table 4:** Validation and regulatory considerations for AI-enabled precision pharmacotherapy.

Framework	Primary Focus	Relevance to Personalized Drug Therapy
FDA AI Guidance	Credibility, transparency, lifecycle management	Regulatory decision support
EMA AI Framework	Trustworthy AI and clinical validation	Clinical implementation
ICH M15	Model-Informed Drug Development	Acceptance of computational evidence
TRIPOD+AI	Reporting prediction models	Transparent model reporting
CONSORT-AI	AI clinical trials	Evaluation of interventional AI
SPIRIT-AI	AI clinical trial protocols	Standardized protocol development
Context of Use (COU)	Risk-based validation	Determines evidentiary requirements
Analytical Validity	Accuracy and reproducibility	Reliable model inputs
Clinical Validity	Prediction of meaningful outcomes	Independent external validation
Clinical Utility	Improvement in clinical decisions	Demonstration of patient benefit

**Future Directions: Digital Twins and Adaptive Dosing**

Although digital twins remain largely investigational, advances in multimodal data integration, computational power, and mechanistic modeling are progressively improving their feasibility for precision pharmacotherapy. In healthcare, a digital twin can be conceptualized as a computational representation of an individual patient that integrates PK, PD, physiological, clinical, and molecular information to simulate potential therapeutic outcomes under alternative treatment scenarios. Unlike conventional predictive models that estimate a single endpoint, digital twins seek to model dynamic interactions among drug exposure, disease progression, organ function, biological response, and treatment interventions over time. In principle, such systems could support prospective evaluation of dose modifications, DDI, organ dysfunction, toxicity risk, and treatment sequencing before therapeutic decisions are implemented. More immediate opportunities are likely to arise from adaptive dosing systems that continuously update therapeutic recommendations as new information becomes available. Current MIPD frameworks already incorporate Bayesian forecasting and patient-specific covariates; however, future systems may integrate a broader range of inputs, including TDM results, longitudinal laboratory trajectories, pharmacogenomic information, medication adherence data, wearable-device outputs, and emerging PD biomarkers. Rather than relying on fixed monitoring schedules and episodic dose adjustment, adaptive dosing frameworks could dynamically reassess therapeutic requirements, such as physiology, disease state, or treatment response changes over time. Such approaches may be particularly valuable in settings characterized by rapidly evolving PK and PD, including critical illness, transplantation, oncology, infectious diseases, and complex chronic disease management. Historically, TDM has focused primarily on achieving target concentrations; however, exposure alone often fails to explain variability in efficacy or toxicity. Emerging biomarker technologies may allow future monitoring systems to directly assess target engagement, pathway activation, adaptive resistance, and early toxicity signals. Integrating biological response measurements with PK monitoring could enable therapeutic optimization based not only on what drug exposure has been achieved, but also on whether the intended biological effect has occurred. This transition may ultimately provide a more mechanistically informed framework for individualized treatment selection and dose adjustment.

**Figure 3:**
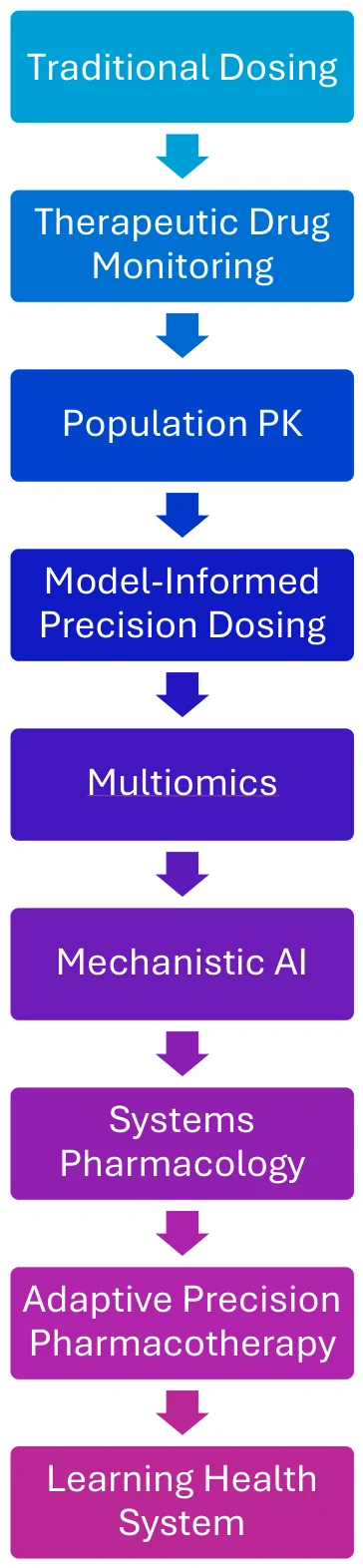
Evolution of personalized drug therapy: From empirical dosing to mechanistically informed precision pharmacotherapy. Conceptual evolution of personalized drug therapy over the past several decades. Initial approaches relied on empirical dosing guided by population averages, followed by therapeutic drug monitoring (TDM) and population pharmacokinetic (PK) modeling to individualize drug exposure. The development of model-informed precision dosing (MIPD) introduced Bayesian pharmacometrics to optimize dosing using patient-specific pharmacokinetic data. More recently, advances in multiomic technologies have enabled characterization of the biological determinants of drug response beyond systemic exposure alone. The integration of mechanistic artificial intelligence (AI) with systems pharmacology represents the next stage in this progression, combining pharmacological, molecular, and clinical information within biologically informed computational frameworks to support individualized therapeutic decision-making. Ultimately, these approaches converge toward adaptive precision pharmacotherapy within continuously learning healthcare systems, where therapeutic recommendations are iteratively refined using longitudinal clinical outcomes, therapeutic drug monitoring, pharmacodynamic biomarkers, and real-world evidence. This progression reflects an evolution from exposure-guided dosing toward mechanism-informed, data-driven optimization of efficacy, safety, and patient-specific benefit–risk.

Future precision pharmacotherapy may increasingly operate within learning healthcare systems. In such systems, routine clinical care continuously generates evidence that improves subsequent therapeutic decisions. TDM results, biomarker trajectories, treatment responses, adverse events, and real-world outcomes become part of an evolving knowledge ecosystem capable of refining pharmacological models over time. Federated learning and other privacy-preserving approaches may facilitate collaborative model development across institutions while maintaining data security and patient confidentiality. If successfully implemented, these continuous learning systems could shorten the interval between discovery and clinical application, improve model generalizability across diverse populations, and support more adaptive, evidence-driven therapeutic decision-making. Ultimately, the long-term objective is not simply more accurate prediction, but the development of biologically informed therapeutic systems that continuously integrate exposure, biological response, and clinical outcomes to optimize efficacy, safety, and patient-specific benefit–risk balance (Figure 3).

## 3. Conclusions

MIPD has advanced this field by introducing Bayesian forecasting, population PK/PD models, and individualized exposure prediction. However, the next step is integration of biological state. Multiomics can characterize the molecular determinants of drug response, including inherited variation, regulatory activity, protein abundance, metabolic phenotype, microbiome effects, and tissue stress. Mechanistic AI may integrate these layers with PK/PD and clinical data to support more interpretable, individualized, and adaptive therapeutic decisions. Platforms such as Operon™ may serve as scientific infrastructure for this transition by connecting heterogeneous biological and pharmacological data into structured, expert-reviewed outputs for development strategy, PK/PD interpretation, off-target risk assessment, and precision pharmacotherapy hypothesis generation. The central challenge is validation. Prospective demonstration of clinical utility, rather than retrospective predictive performance alone, will ultimately determine whether these technologies become routine components of personalized pharmacotherapy. AI-augmented TDM, digital twins, and multiomic precision dosing will only become clinically meaningful if they demonstrate prospective improvement in target attainment, therapeutic response, safety, cost-effectiveness, and equity. The field must therefore move beyond retrospective discrimination toward calibrated, interpretable, context-specific, and outcome-linked evidence. Successful clinical translation will ultimately depend not on increasingly sophisticated algorithms, but on rigorous prospective validation demonstrating that AI-guided precision pharmacotherapy measurably improves efficacy, safety, and patient outcomes beyond existing standards of care.

## Figures and Tables

**Table 1:** Limitations of conventional therapeutic drug monitoring and opportunities for AI-augmented precision pharmacotherapy.

Limitation of Conventional TDM	Clinical Consequence	Opportunity for AI-Enabled Precision Pharmacotherapy
Drug concentration is an imperfect surrogate for pharmacological effect	Similar concentrations may produce different responses	Integrate PK with PD biomarkers and pathway activity
Sparse sampling and delayed laboratory results	Misses rapidly changing physiology	Adaptive monitoring with continuous learning
Focus on a single analyte	Ignores systems biology and disease mechanisms	Integrate multiomic biomarkers and systems pharmacology
Drug-centric rather than regimen-centric	Underestimates cumulative toxicity and DDIs	Model regimen-level interactions using network analysis
Population-derived therapeutic ranges	May not reflect individual biology	Patient-specific Bayesian and AI-guided dosing
Limited integration with longitudinal clinical data	Static rather than dynamic decision-making	Real-time updating using EHR and wearable data

**Table 2:** Contributions of multiomic technologies to precision pharmacotherapy.

Multiomic Layer	PK Applications	PD Applications	Toxicity Assessment	Representative Clinical Utility
Genomics	Drug metabolism enzymes	Target variants	Pharmacogenomic risk	Initial drug selection
Epigenomics	Enzyme regulation	Adaptive responses	Treatment resistance	Dynamic disease characterization
Transcriptomics	Enzyme/transporter activity	Pathway activation	Early molecular response	Monitoring biological activity
Proteomics	Transport proteins	Receptor abundance	Organ injury proteins	Pharmacodynamic biomarkers
Metabolomics	Functional metabolism	Cellular metabolic state	Mitochondrial toxicity	Early toxicity detection
Lipidomics	Membrane transport	Inflammatory signaling	Lipotoxicity	Disease endotyping
Microbiome	Drug metabolism	Immune modulation	Altered exposure	Personalized dosing
Extracellular Vesicles	Tissue-specific biomarkers	Target engagement	Organ-specific injury	Longitudinal monitoring

**Table 3:** Comparison of complementary computational approaches for personalized drug therapy.

Characteristic	Predictive AI	Mechanistic AI	Systems Pharmacology	Model-Informed Precision Dosing
Primary objective	Predict outcomes	Explain biological mechanisms	Model biological networks	Optimize drug dosing
Principal inputs	Clinical datasets	Clinical + biological knowledge + multiomics	PK, PD, molecular pathways	PK, covariates, concentrations
Principal output	Risk prediction	Mechanistic inference	Systems-level response	Individual dose recommendation
Biological interpretability	Low–Moderate	High	High	High
Typical methodology	Machine learning	Knowledge-constrained AI	Mathematical models	Bayesian pharmacometrics
Clinical application	Prognosis	Precision pharmacotherapy	Drug development	Therapeutic drug monitoring
Major limitation	Limited biological explanation	Requires validated biological knowledge	Model complexity	Limited biological context

## Data Availability

No new data were created or analyzed in this study. Data sharing is not applicable to this article.
